# Sexual and physical abuse and its determinants among street children in Addis Ababa, Ethiopia 2016

**DOI:** 10.1186/s12887-018-1267-8

**Published:** 2018-09-19

**Authors:** Ayana Chimdessa, Amsale Cheire

**Affiliations:** 1 School of Nursing and Midwifery, College of Health Sciences, Wollega University, P.O Box: 395, Nekemte, Ethiopia; 2 College of Health Science, Addis Ababa University, Addis Ababa, Ethiopia

**Keywords:** Street children, Sexual and physical abuse, Drug use, Addis Ababa, Ethiopia

## Abstract

**Background:**

The life and health of street children is becoming a global concern. Street children are vulnerable to a variety of problems including physical, psychological and sexual exploitations as well as social isolation. Therefore, it was the purpose of this study to point out the experience of sexual and physical exploitation and its determinant factors among street children in Addis Ababa, the capital city of Ethiopia.

**Methodology:**

A phenomenological qualitative method was conducted from March to June 2016 in Addis Ababa, Ethiopia. Data were collected from street children by focus group discussion (FGD) and in-depth interview. Open code was used to analyze data. The transcribed note was translated. Following this coding was done. Based on a coding book, major themes and main categories were developed and analyzed.

**Result:**

The study has explored the life experience of street children in the city. Deaths of parents/unhealthy relationship of extended families forced them be on a street. Thus, flee to street to search for work was the main reason for joining to a street. Street children are vulnerable to sexual and physical exploitations on a daily basis. For street children, street is the world characterized by misery deprivation, physical, verbal and sexual abuse and become daily victims of violence. There is no safe place for the children of on-street. Most street children are involved in all types of sex; heterosexual, bisexual, homosexual and group sex are common among themselves and out siders. They are involved highly in transactional sex for survival. Drug use, stress and depression are common experiences. Thus, they were socially isolated. The main challenges of living on a street are lack of basic needs, social isolation, lack of safety and security and being out of school are the common problems these vulnerable groups are facing.

**Conclusions:**

Street children are at high risk of sexual and physical exploitation. Interventions targeting integration and reunion with families, reduction of physical and sexual exploitation, access to education, mental health promotion and reduction of drug use behavior should be taken in to considerations.

**Electronic supplementary material:**

The online version of this article (10.1186/s12887-018-1267-8) contains supplementary material, which is available to authorized users.

## Background

The life and health of street children is becoming a global concern. Street children are vulnerable to a variety of problems including physical, psychological, sexual and social isolation. Children who spend time on the street are at risk of increased aggression, hopelessness, drug use, and informal sex work [1].

Though street children are hard to count, a recent global estimate showed that nearly 150 million street children are living in the urban and semi-urban areas [2, 3]. The number of street children is increasing worldwide for various reasons [3]. Factors related to family disruption and lack of basic needs has been strongly associated with these marginalized groups of people [2, 3]. HIV/AIDS is the most significant global factor to increase the number of orphans, particularly in sub-Saharan Africa. Studies indicated that there were 17.3 million orphans due to HIV/AIDS globally. Out of these, the majority of orphan and vulnerable children (OVC) are found in sub-Saharan Africa and Southern and Southeastern Asia [4]. Children who lost their parents are at increased risk of physical, psychological, social, health, and economic problems. Lack of parental protection, guidance and support may predispose children to street life and made them highly vulnerable group [5–7].

In Ethiopia, over 4 million children are estimated to live under difficult circumstances of poverty and health problems. It is estimated that 600,000 children are taking part in street life. As many as 500,000 children are at high risk of becoming involved in street life in Ethiopia [2].

Studies conducted in different part of sub-Saharan countries showed that street children are one of the high risk groups to acquiring sexually transmitted infections including HIV/AIDS. They are prone to sexual abuse, rape, prostitution, sexual bartering and exchange, casual sex and early exposure to both heterosexual and homosexual behaviors [1, 5, 8].

Thus, exploring the magnitude of sexual and physical exploitation among street children is a pivotal to address the needs of street adolescent and mitigating the negative outcomes of the growing street children population worldwide. Therefore, it was the aim of this study to assess the experience of sexual and physical exploitations among street children in Addis Ababa, Ethiopia.

## Methods

### Study design and period

The study was conducted in Addis Ababa, the capital city of Ethiopia using phenomenological qualitative study design. All on and off street children age 10–18 years old were involved in the study. The phenomenological inquiry consisted of in-depth interview and focus group discussion (FGD) for both on and off street children. The study was conducted from March to June 2016.

### Sample and sampling techniques

Snowball and purposive sampling strategies were employed in recruiting participants into the study. Both FGD and in-depth interview were conducted up to the level of information saturation. Both on and off street children were participated in FGD and in-depth interview. Totally six FGDs each consisting of 4–10 members (*n* = 41) and in-depth interviews (*n* = 20) were conducted.

### Data collection procedure

Semi- structured FGD and in-depth interview guides (Additional file 1) were used to explore the life experience of study subjects. Both FGD and in-depth interview lasted for an average of 60–70 min. Study subjects were separated into two based on their sex in order to get more insight of physical and sexual experience as well as factors affecting it. All FGDs and in-depth interviews were conducted in a private place for the purpose of concentration and privacy of participants. Permission to use a tape recorder was obtained from the participants prior to actual data collection. Six trained BSc nurses and two supervisors who have experience in qualitative data collection have taken part in data collection.

In this study**,** children off-street were those who were working or begging on street but, either living with their parents/relatives or visiting their parents/relatives regularly. Children on the street were those who live and work on a street without any kind of supervision from parents or relatives. They were both economically and socially dependent on a street life.

### Data processing and analysis

Data inquiry was analyzed using content analysis software. The transcribed note was translated. Following this coding was done. Based on a code book, major themes and main categories were developed following the research objectives and questions of the study. Researchers identified the major themes and main categories of the research. After the themes and categories are identified intuiting the data was done. Analysis of data includes coding, categorizing and making sense of the essential meanings of the phenomenon. Researchers came to understand and describe the phenomenon. Results were written by reorganizing, summarizing and quoting when needed.

Ethical clearance was received from Addis Ababa University. A formal letter was written to all concerned authorities and permission was secured at all levels. Since the study subjects were younger than 18 years, written assent was taken from study subjects and written consent was taken from guardians or nongovernmental organizations (NGO) who were responsible to look after them. To compensate the study participants’ time, the researchers provided them 15 birr (USD 0.5) per person. Participation in the study was voluntary and information that was collected from the study subjects assured confidentiality.

## Results

### Demographic characteristics of study participants

In this study, snowball sampling strategy was employed in recruiting the study participants. Data collectors interviewed available on and off-street children throughout the data collection period which resulted in different numbers of male and female study participants. Thus, a total of 40 on-street and 21 off-street children aged 10–18 years were participated in the study. Of which, 46 were male and 15 were female street children. Among these (*n* = 61), 20 street children (on-street *n* = 12) and (off-street *n* = 8) were participated in the in-depth interview. Whereas, a total of 41 street children from both categories (on-street *n* = 28) and (off-street *n* = 13) were involved in the six FGDs.

During data collection period, data collectors had registered participants who were in and out of school on their field notes. Based on the field note memo the number of children in and out of school were well noted. Of the total study participants (*n* = 37) had never been to school, (*n* = 9) were dropped their schooling and (*n* = 15) were in school during data collection period. The finding showed that there was a huge gap of schooling between on and off-street children. The result shows that the number of off-street children who were in school was higher than on-street children. Out of 40 on-street children about (*n* = 31) of respondents had never been to school. Whereas, out of 21 off-street children (*n* = 6) of them had never been to school during data collection period. About (*n* = 9) of on-street children reported that they were dropped their primary education. Whereas, (*n* = 15) off-street children were in primary education during data collection. Out of 40 on-street study participants about (*n* = 12) of them were informally married to one another in order to cope with the stressful street life. However, all off-street children were reported never married (Table 1).

**Table 1:** Demographic characteristics of street children in Addis Ababa, Ethiopia 2016

Research tools used	Category of study participants	Number of participants	Sex		Educational level			Marital status	
			Male	Female	Never been to school	Grade 1–6	Grade 7–8	Single	Informally married
FGD									
FGD 1	On street children	7	7	–	6	1	–	4	3
FGD 2		9	9	–	7	2	–	7	2
FGD 3		8	8	–	7	1	–	5	3
FGD 4		4	–	4	3	1	–	3	1
	Total	28	24	4	23	5	–	19	9
FGD 5	Off street children	5	–	5	1	2	2	5	–
FGD 6		8	8	–	2	5	1	8	–
	Total	13	8	5	3	7	3	13	–
In-depth interview	On street children	12	9	3	8	3	1	9	3
	Off street children	8	5	3	3	3	2	8	–
	Total	20	14	6	11	6	3	17	3

A total of 41 discussants participated in FGD: on-street (*n* = 28) and off-street (*n* = 13)

A Total of 20 children participated in in-depth interview: On-street (*n* = 12) and off-street (*n* = 8)

### Determinants of being on street, life on street and challenges of being a street child

The findings of in-depth interview and FGD on the factors that exposed children to street life, their life experiences and challenges they were facing by being on street is shown in Table 2.

**Table 2:** Major thematic areas and main categories of in-depth interviews and FGDs of street children in Addis Ababa, Ethiopia 2016

Theme	Categories
Theme 1: Determinants of being street children	1.1. Death of parents and other relatives 1.2. Searching for work
Theme 2: Context of living on a street	2.1. Sexual practice: Early initiation of sex, coercive sex, transactional sex, heterosexual, bisexual and homosexual sex, nonuse of condom, multiple partners 2.2. Drug use behavior and mental illness 2.3. Involving in income generating activity 2.4. Low knowledge of STIs and HIV
Theme 3: Challenges of living on a street	3.1. Lack of basic needs, social isolation and lack of safety and security 3.2. Being out of school

### Theme 1 determinants of being street children

Participants pointed that there are different factors to practice street life. Out of these, respondents have given stress to factors like death of the family, extended family relation destruction and poverty. After the death of their parents, they were being on street or flee to extended family to search for daily basic needs. From their experience participants revealed that most of the time they flee to extended family. If the relation with their extended family become unhealthy, they would be forced to join a street life. The other factor forced them to join a street life was poverty. Respondents indicated that when their family face challenges of poverty, unable to educate and cloth their children, the children flee from home and search for jobs in the near towns. Later fled to more industrialized and urbanized cities.

#### Category 1.1: Death of parents and other relatives

The deaths of parents were one of the main reasons forcing them to street life. Discussants said that most of them joined street life after the death of one or both of their parents. They revealed that “*if you don’t have parents you will not have any one to fulfill the daily basic needs*. *You don’t have anyone to take care of you, to support you, to protect you and to love you”* (FGD 1, 3).

Interviewees pointed that death of their mothers was more severe and have affected their life significantly. The interviewees pointed their reason of being on street was; “*following my mother’s death my father married another woman. This step mother was not friendly, she was unloving, she insults and beats me every day and she was not giving me enough food. Because of this I run away. I joined a street life”* (Interview 5, 7, 19).

The FGD discussants and other interviewees also shared the same experiences. They said loss of parents was the main reason for a considerable number of children to be on street. “*If your parents die, you will lose everything: parental love, means of survival, shelter and security. You and you alone, you have to struggle to survive by using all means you know, like surviving through scavenging garbage and sharing experience from friends how to survive street life”*(FGD 5 and interview 10, 14).

#### Category 1.2: Searching for jobs

Focus group discussants reported that most street children drifted to Addis Ababa and other big cities to search for a job. Some of the street children migrated to Addis Ababa hoping that they could get a better job to change their life unfortunately, the situation was quite different. What they experienced was not what they dreamed and expected. Participating in manual work was common practice. *“Doing this manual work with empty stomach and beyond your age was sometimes difficult to bear. Then you will be involved in any other activities that helps you to get your daily basic needs*” (FGD 1, 6).

### Theme 2: Context of living on street

Participants reported that context of living on street was hard to survive. Sexual activity among street children was common practice to survive this harsh life. On daily basis, these situations exposed to risky sexual behaviors and positioned them under various health problems.

#### Category 2.1: Sexual practices among street children

Street children were highly involved in risky sexual behaviors in order to survive. These situations exposed them to sexually transmitted infections (STIs) and HIV/AIDS.

This study shows that street children articulated experiencing sexual exploitation that could predisposed them to various health problems. For the street children, environment does not provide protection against such vulnerability. The study also revealed that new arrivals on streets especially girls are used as sex objects by older boys and watchmen of shops at night. Mostly, such sexual relationships were for protection to the newcomers. In the case of those newcomers that refused such sexual advances, they were beaten up and chased away from that particular area of the street. From the six groups of FGD two groups reported that they have a girl friend or wife; which is an informal arrangement made by the homeless street children. However, being under informal agreement, their wives used to go for sex work with strangers to get money and comes back to her informal husband **(FGD 4 and 2)**.

##### Transactional sex

All discussants pointed that street children participate in risky sexual behaviors on daily basis for their survival. They reported that if they do not involve in survival sex, living on street will be unbearable. They indicated that no one is there to provide them with basic needs like food, cloths rather than dying from hunger. “***We sell flesh to survive on street”*** **(FGD 1 and 2).**

##### Condom use

Participants reported that inconsistent and nonuse of condom were common practices among street children during sexual intercourse. Interviewees said that condom usage depends on their partner’s interest. Most partners particularly, strangers prefer to have sex without condom. Respondents said that “***we were unable to negotiate to use condom with strangers instead of doing what they want and get money for our survival. We obey to our partners’ interest/decisions to survive on street”*** **(Interviewees 1, 4 and FGD 3)**.

##### Types of sex

The study revealed that sexual intercourse among street children and outsiders were common. The discussants articulated that ***“most of us are involved in all types of sexual intercourses: heterosexual, bisexual, homosexual and group sex were common practice among us and outsiders”*** **(FGD 2 and 4)**. There is no safe place for children of on-street. Study participants indicated that no safer places to spend the night, both boys and girls sleep in the same quarter***.*** The focus group participants pointed that all types of sexual relation does exist on a street “***Sexual relationship among street children and with outsiders is widespread. Thus, multiple partnership is a common practice”*** **(FGD 2, 3, 6).**

##### Coercive sex

Forced sex was common within a street group and from outsider communities. Interviewees pointed that the most difficult challenge they were facing was older street children forces the younger ones to have either vaginal or anal sex. “***We street children have no one to stand for us. As far as you are in the loop, you cannot escape except, accepting whether forced homosexual or heterosexual and even physical and sexual misuse. You don’t have any one to protect you. For whom would you cry?***” **(interviewee 5, 8, 19).**

Two groups indicated that ***“we try to defend ourselves from unwanted sex with strangers by sleeping in a group. If we get a job and paid money, we may spend the night at the bed room called DC (low price and poorly cleaned hotel around Merkato) which we have been paying 15 birr (USD 0.5) per night. Since it was difficult to get money to spend the night at DC we sleep in a group to protect ourselves from strangers over the night*.** ***Our dogs are also with us every night. If somebody came to hurt us at night our dogs alerts us”*** **(FGD 1 and 4)*.*** The study also revealed that female street children who spend a night over street face another challenge called **‘gelbo’. “**Gelbo” means in their language female street children being a victim of forced sex while sleep on a street during night time **(FGD 4, 6).**

#### Category 2.2: Drug use behaviors and mental illnesses

Living on a street had direct relation to being exposed to drugs. Participants pointed that ***“life on street is misery***. ***You feel hopelessness, your current and future life is dark. We cry out for help but from where? There is no one by our side. At times we feel lonely, suffer from lack of sleep, anxiety, isolation and face mood depressions. To subsist all these; we use variety of substances: like glue, hashish, cannabis, khat and local alcoholic drinks such as arekie and tej” (FGD 1, 4, 5, 6).*** Discussants indicated that the use of substances helps them to temporarily relieve themselves from the hurdles they were facing on a street. They said “***Even dogs and cats have their shelter but, we …we have to hide ourselves from our selves by taking different substances. We use variety of substances to temporarily mask our problems and relive from the pain of it. By taking these substances, we enjoy the temporary pleasure of it and forget the disgustful life we are facing on a street” (*FGD 2, 3, 5 and 6) and interviewees (12, 13, 17 and 19).**

#### Category 2.3: Engagement in income generating activities (IGA) on a street

Street children get basic needs through different ways. The study participants pointed that they were generating income through begging, participation in informal sex work, survival sex and manual work. These all activities were the main income generating methods to subsist.

##### Begging

Begging on a street was one of the strategies to survive. Discussants pointed that “***Although we don’t like it, we beg to get our daily bread. This is our day to day activity. If we have a family or anybody who care for us, rather than begging by this age; we prefer to go to school”*** **(FGD 2, 6 and interviewee 7, 11).**

##### Transactional sex/survival sex

All study participants extensively discussed that transactional sex was the major income generating activity in easy approach. Interviewees pointed that “***We sell our flesh just to survive”*** (**Interviewees 8, 13, 20).**

An interviewee stated that “***you don’t have option rather than living this ugly life; I had anal sex with strangers in exchange of food and other goods. If my parents were alive this would not happen to me. But I have to live this way and face the ugly life for subsistence”.*** Some discussants and interviewees shared the same experiences **(Interviewees 3, 7, 15).**

Out of the six groups, two groups reported “***The other most intricacy we street children facing is; rich people who have cars come and talk with us, give us cloth, food, money or other goods and takes us to their home. After you reach home, they force you to have sex with them”*** **(FGD 1, 3).**

There were countless street children who were forced to have either anal or vaginal sex with strangers. One focus group participants cited one example: “***one day one of our friend was taken by a rich person who had been driving his own car to his home. After our friend reached home, the man forced him to have anal sex. Although I cited this example, it is a common experience of numerous street children”*** **(FGD 1).**

Three of the interviewees also expressed their deep sorrow with their eyes filled of tears: ***“You don’t have right on yourself; you simply permit those who have money and power to play on your flesh. You allow them to do whatever they want because they have money. You need to survive. This is what happens to me in most instances”*.** Other interviewees shared similar sorrow in their life **(Interviewee 2, 3, 18).**

##### Manual work

Street children use different mechanisms to survive on a street and at the same time their life style endanger their health. The other issues that exposed street children to health problems was participating in heavy manual work in order to survive on a street. They participate in heavy manual work in exchange of breads or cloths in daily basis.

In this study, participants cited that “***we participate in sifts through garbage in order to collect recyclable materials such as plastics, paper and metal, cleaning cars, petty vending, selling small items, shining shoes, digging, carrying heavy loads which compromise our health and schooling. We usually rise before sunrise and carry our heavy load by bag over our shoulder and change it with basic needs like bread, money and cloths”*** **(FGD 3, 5).** All study participants, both on and off-street children share the same experience of manual work.

#### Category 3.4: Knowledge of STIs and HIV/AIDS

Misconception about STIs and HIV transmission and prevention was widespread among street children. Study participants said that they heard about STIs and HIV/AIDs. However, discussants had misconceptions about transmission and prevention of HIV. Most of them responded that HIV can be transmitted by lice, sputum expectoration, and drinking water with unclean or used cup. Most of participants cited that “***walking on bare foot is one of the way to transmit HIV***” **(FGD 2, 5).**

### Theme 3: Challenges of living on a street

Respondents were glowing up that they were facing different challenges while they were living on a street.

#### Category 3.1: Lack of basic needs, safety, security and social isolation

The challenge of living on a street was described by discussants as: “***Street is our home, it is our social abode. We live in the streets and eat on streets. Street life means hunger, deprivation of basic necessities, being homeless, sleeping on the ground, passing the day on the sun and suffering from the cold at night”. “We face begging, physical and sexual exploitation on the daily basis. Living on a street means lack of belongingness, being neglected, lack of protection, discrimination, social exclusion, drug addiction, and participation in prostitution”*** (**FGD 1, 2 and interviewees 1, 7).**

All categories agreed that the most challenges of living on street were “***hunger, cold and rain. In addition to this, police men consider us a pickpocket person and abuse us physically every day”*** **(FGD 4, 5, 6)**. Streets children were victimized on a daily basis. Focus group discussants pointed that police men consider them as criminals/ pickpocket person and strike them without any reason and had been taking their things (jalbo). As participants indicated sometimes police men had been taking them to jail without any legal background. “***Street children are not criminals rather than people who live with their own problems on a street*.** ***I am facing different challenges by being on street: hunger, cold, sleep on a street while flood flows under the place where I sleep on a street”*** **(FGD 5, and Interviewees 12, 17, 20**)***.***

Female participant revealed that the other most challenge of living on a street was “***suffering of rain during rainy season; there is no any roof to sleep under and no enough cloth to wear. Imagine, the hunger you face during rainy time! Sleeping on a street with the agony of hunger since you do not get enough food is another hurt we are facing on a street***” **(Interviewees 16, 18).**

#### Category 3.2: Being out of school

On-street children have not got any opportunity to attend their educational endeavor. To attend the school basic needs are obligatory. Thus, they devoted their time in searching of basic needs rather than attending school.

Most of children on-street never attempt to join school due to lack of school requirements, food and other basic needs. They had nobody to support them for their schooling. Those who attempt school, forced to drop their school due to lack of money for school requirements such as school fees, uniform and shoes. Incapability to concentrate to attend school due to hunger. Thus, regular absenteeism from the school results in the loss of interest to continue their education was another tricky situation that these young vulnerable groups were facing.

Participants’ highlighted that ***“Living on a street means being out of school***. ***We are created simply to watch other children who have parents, home and going to school. Even if we want to go to school, we can’t. We don’t have the pre requisite for learning. It is difficult to be enrolled, to get teaching materials, to be accepted by other students in the school. We blame our hard luck”*** **(FGD 1, 3).**

Off-street children reported that ***“We have opportunity to be in school when we compare ourselves with our colleagues. However, absenteeism from school is the challenge we are facing. To survive you should search for daily manual work” (FGD 5, 6).***

## Discussion

This study provided the opportunity to look at the life experience of street children related to determinant of being on street, context of living on a street and challenges of living on a street of this young vulnerable group in Ethiopia, Addis Ababa city.

The findings of this study revealed that death of parents (single or double orphan) or conflict with extended relatives were determinant factors to be street child. Thus the death/conflict with extended family resulted in poverty. As the consequence, they were forced to participate in transactional sex and risky sexual behaviors. Lack of basic needs was perceived to lead to engage in survival sex. It is strategy to meet such need. This finding is similar with the study conducted in Malawi and Kenya [3, 9].

This phenomenological inquiry revealed that socio-economic factor puts street children at elevated risk of sexual behaviors. To resist the economic problem, street children initiate early sexual engagement. Thus, multiple sexual partners is perhaps in their life. This is consistent with the study conducted in Liberia and Uganda [10, 11].

The result shows prominent report on context of living on a street. The context is full of risky sexual behaviors, early sexual initiation, multiple sexual partners and physical exploitation were common practices. The finding of this study is similar with the study conducted in sub-Saharan countries [9, 12, 13]. The finding revealed that street children have been found to be at higher likelihood of being sexually active, initiate sex at an earlier age, have unprotected sex and multiple partners. In this study participants reported that street children are involved in high risk sexual behaviors every day for survival. The studies conducted in southern Africa, Uganda and HIV report on global have documented higher rates of STIs and HIV/AIDS among street children [9, 14–16].

This study revealed that street children are facing sexual abuse**,** rape, prostitution, sexual bartering from day to day which is the same trend with the study conducted in sub-Saharan African countries [12, 14].

This finding shows that street children are high risk group for STIs and HIV. Both male and female street children spent their night at same domicile. Being out of school and insecurity life on a street are the challenges they are facing in their day to day life. Thus, it makes sexual relationships wide spread among themselves and with outsiders. This is similar with the study conducted in Malawi and other sub-Saharan countries [3, 12, 16].

This report shows that street children are using different substances to be out of depression and mood fluctuation. Inline to this finding, the study conducted in south Africa shows (30.3%) of street children had reported clinical depression [14].

There was poor knowledge of STIs and HIV/AIDS among street children. Similarly, different studies shows that respondents’ knowledge about STIs and HIV/AIDS is very low [10, 15].

## Conclusion

The study has explored the life experience of street children. In different aspects street children are vulnerable to sexual and physical exploitations on the daily basis. The study verified that street children are the most vulnerable group of young people that their life style put them under the circuit of risky sexual behaviors, sexual abuse and physical exploitations.

Rapes, anal sex, unprotected sex and survival sex are common factors that increase healthy problem of them. On-street children has no safe sleeping place over the night. Thus, street environments do not offer protection to them from sexual and physical abuse. Lack of safe night shelter places street children on high risk of sexual and physical exploitations and added an advantage of prostitutes for strangers. This study showed that children off-street is less likely to be sexually exploited compared to children on-street as they share night shelter with their families or extended families.

## Recommendations

Physical and sexual exploitation reduction programmes targeting both on-street and off-street children should be taken in to considerations through multi-cultural and community involvement perspectives. Creating income generating activities should be considered for these young vulnerable groups. Creating social protection systems under the collaboration of governments and non-government organizations should be considered. Forming temporary residency and try to re-unite with their families has sufficient advantage in their healthy. Not only for their health, has indisputable credibility to avoid social isolation. Additionally efforts should be targeted to increase street children schooling and opportunity for safe shelter (separate quarter for male and female street children) over the night. (Government organizations and NGOs like Hope Enterprise should work on it).

The researchers recommend mega researches on both on and off-street children that can identify their STIs and HIV/AIDS statuses by using large sample size. Large sample size that allow mixed qualitative and quantitative researches recommended to understand the life experience of street children in different cities of Ethiopia.

### Limitation of the study

This study used only phenomenological qualitative research design that used small number of study participants up to the level of information saturation from a single city.

## Additional file

Additional file 1: Data collection tools. (DOCX 25 kb)

## Abbreviations

BSc: Bachelor of Science
FGD: Focus Group Discussion
IGA: Income Generating Activities
NGO: Non-Government Organization
OVC: Orphan and Vulnerable Children
STIs: Sexually Transmitted Infections
