# Supplementary material for: Sexual and physical abuse and its determinants among street children in Addis Ababa, Ethiopia 2016
Source: BMC Pediatr. 2018 Sep 19;18:304. doi: 10.1186/s12887-018-1267-8 (PMC6146752; doi:10.1186/s12887-018-1267-8)
Supplement: Supplementary file 1 — Data collection tools. (DOCX 25 kb) [file 12887_2018_1267_MOESM1_ESM.docx]

***Brief information for focus group and in-depth interview study participants.***

***Hello, my name is ________. I am data collectors of the research entitled sexual, physical abuses and its determinants among street children. The goal of this research is to better understand sexual, physical abuse and its determinants among street children. This research finding will help to identify sexual, physical abuse and its impact on the health of street children. The finding will help to mitigate the negative outcomes of this underprivileged group over worldwide. Therefore, it is the aim of this research to assess the experiences of sexual and physical exploitations of street children. We hope this research finding help the government, non-government organizations and policy makers to develop effective interventions to improve the health of this underprivileged group.***

***You have been invited to take part in the study because you are living on a street. Your opinion and perspective will help us to identify the barriers to your health. If you agree to participate today, we will ask you to sign the assent form and we collected the consent form from your guardians or organizations who were responsible to look after you. You will participate in a focus group discussion or individual in-depth interview with trained data collectors. To get your experiences of a street life, we separate male and female participants group. The activity will meet once for 50 to 60 minute and you will be provided with a copy of assent form. If you have any concern, you have the right to ask your questions at any point.***

***You can choose not to take part in the study and if you join, you can choose to quit at any time. There will be no penalty to you, your supporters or your community and we will not be offended. There will be no penalty for skipping questions or deciding you want to end your participation.***

***The primary benefit of participating in the study is that the finding will be used to further improve the care and support by available services to street children in Ethiopia and beyond. Additionally, to compensate your time the researchers will provide you 15 birr (USD 0.5) per person. Participation in the study is voluntary and information that will be collected from you will assured confidentiality. For FGD participants: At the beginning of the focus group session, the group will create a mutually agreed upon code of conduct that covers issues of confidentiality and ethics within the group. The results will be shared with national stakeholders and we writes scientific reports and articles for sharing of the findings.***

***Note for all data collectors:***

***Dear data collectors,***

***Please do not forget to take a note about socio- demographic characteristics for each of study participants (both FGDs and in-depth interviews) on your field note memo. It should include code, category of study participants (On or Off-street children), age, sex educational back ground, current schooling status (in school or dropped out), marital status (single or informally married), ethnicity, etc.***

**Questioning Guide for Individual In-depth Interview of Street Children**

**Part I: General background information of interviewees**

1. Were you born here in Addis Ababa? If no, where did you come here? For what reason did you flee from your home?
2. What happened to you on your first arrival to this city? Who helped you during your first arrival to this city?
3. What things did you do on your first arrival? Please describe all actions you have taken.
4. What made you a street child? What major problem put you on a street? (Probing: death of family, poverty, destruction of extended family relationship, search for job etc.)
5. If it was death of a family/ parent/s, was there any anxiety, depression and mood disorder within the past 12 months related to the death of your family?
6. Are you attending school? If yes, who sponsored your education? (Individuals, groups, organizations, community etc.) If you are not attending or dropped schooling, why?
7. Have you been ill while living on street? If yes, have you gone to a health institution? If yes, who have taken you to health care institutions? If no, why?
8. Looking at your history, can you tell us about your health and lifestyle experiences of street life?
9. When you join this street life, how did you socialize with other street children? What measures have you taken to familiarize yourself with others? Please describe.
10. Where do you spend over night? Is there any separate place for girls and boys?
11. How do you protect yourself/ yourselves when you are living on street?
12. What are the major challenges you are facing by being a street child?
13. Do you use drugs? If yes, which type of drugs? (Probing: khat, marijuana, glue, hashish, benzene, local alcohol drinks like Araqe, tella, teji etc.) For what reasons do you use these drugs?

**Part II:** This part is the interview guide to know your Knowledge about STIs and HIV/AIDS. To continue this part again we ask your permission for the tape recording. Again your responses are confidential in whatever and where ever.

1. Have you ever heard of STIs and HIV/AIDS? If you had the information about it, from where did you get the information?
2. What are the major means of transmission of STIs and HIV/AIDS and how can you protect yourselves from them?
3. Do you know diseases that can be transmitted through sexual intercourse? If yes, please list them.
4. Can you describe symptoms of STIs? Have you ever had any of these symptoms within the past 12 months?
5. If yes for Q 4, did you receive any treatment? If yes, how and where did you get the treatment? If not, why?
6. Have you ever checked your health status for sexually transmitted diseases? If not, why?

**Part III:** This part is the in- depth interview guide to know your sexual practices starting from the time you started street life. To continue this part again we ask your permission for the tape recording. Again your responses are confidential in whatever and where ever.

1. Have you ever had sexual intercourse? If yes, with whom? At what age have you started sexual intercourse? What are the benefits you get from your partner/s to have sex or to be initiated?
2. While you have started or having sex, do you have a full interest and/or an autonomous decision to have a sex with your partner/s? If no, what forces you to have sex with him/her?
3. What methods did you use or have you been using to protect yourself from STIs and HIV/AIDS? If not, why? Please describe.
4. What methods did you use or have you been using to prevent pregnancy? If not, why? Please describe.
5. Who was your first sexual partner? How did you meet him or her? Where did you meet her/him?
6. What were your reasons for you to have a first sexual experience?
7. How frequent do you have sexual intercourse with in a week? How many partners do you have or with how many peoples you had sex so far?
8. We would like to know, how you get basic needs? (Probing: food, shelter, school fee, cloth, shoe and the like).
9. What is/ are the major sources of income to sustain your daily life? (Probing: survival sex, sex exchange, manual work, shoe shine etc.)
10. Do you think you are at high risk of forced sex? If yes, what are sources of those pressures?
11. Have you ever given/taken anything from your partner/s to have sex with him/her? If yes, what have you given/ taken?
12. Is there your partner/s willingness to use condom/ barriers when you have sex with him/her? If no, why?
13. Do you think that you are liable/ vulnerable for high risk sexual behaviors? Why? (Probing: among street children, outsider community, strangers, group sex, homo/ heterosexual, oral/anal sex etc. Please describe.
14. Do you have anything to add/ say more about your street life experiences?

**Questioning Guide for Focus Group Discussion of Street Children.**

1. Were you born here in Addis Ababa? If no, from where did you come from? For what reasons you came here?
2. What made you a street child? (Probing: death of family, poverty, destruction of extended family relationship, search for job etc.)
3. What does a street child/ children mean? What are your survival methods of street life?
4. How do you see sexual relationship among street children and with outsiders?
5. Do you know about sexually transmitted diseases? If yes, what do you know?
6. What about types of STIs? Please list some of them. What are the protection/ preventive mechanisms and how can you protect yourself from sexually transmitted infections?
7. How can you fulfill/ secure your basic needs like food, school fee, cloth, shoe and others?
8. Is there any transaction including items or goods like drugs, money, food or others among street children or outsiders for having sexual intercourse?
9. Where do street children spend over night?
10. What are the major challenges of being a street children?
11. How do you protect yourselves from any criminal attack while you are on street?
12. We would like to know more about your experiences of life difficulties by being street children? Can you describe measures you take or have been taking to overcome those life difficulties?
13. Do you think that high risk sexual behaviors take place among the street children and outsiders? If yes, what kinds of risky sexual behaviors take place?
14. How do you see street life versus drugs?
15. How about you? Do you use drugs? If yes, which type of drugs? (Probing: khat, marijuana, glue, hashish, benzene, local drinks like Araqe, tella, teji etc.) For what reasons do you use these drugs?
16. Do you have anything to add or think to be included or raised about street life in this study?

Dear participant/s,

I thank you!
